# Antimicrobial resistance and genomic characteristics of *Salmonella* from broilers in Shandong Province

**DOI:** 10.3389/fvets.2023.1292401

**Published:** 2023-11-23

**Authors:** Liyuan Zhao, Gang Liu, Wenli Tang, Xiangbin Song, Xiaoyu Zhao, Chu Wang, Youzhi Li, Ming Zou

**Affiliations:** ^1^College of Veterinary Medicine, Qingdao Agricultural University, Qingdao, China; ^2^Shandong Provincial Key Laboratory of Quality Safety Monitoring and Risk Assessment for Animal Products, Shandong Center for Quality Control of Feed and Veterinary Drug, Jinan, China

**Keywords:** *Salmonella*, WGS, antimicrobial resistance, broilers, resistance genes

## Abstract

**Introduction:**

The emergence of multidrug-resistant (MDR) strains of *Salmonella,* which is a genus of important zoonotic pathogens, has aroused great public health concern worldwide.

**Methods:**

In this study, 167 strains of *Salmonella* were isolated from 947 samples from broiler farms, slaughterhouses, and markets in Shandong Province. Antibiotic sensitivity testing was performed, and 70 strains of *Salmonella* were screened out by whole-genome sequencing (WGS) to evaluate serotypes, antimicrobial resistance genes (ARGs), the prevalence of class 1 integrons, the plasmid carriage rate, and phylogenetic characteristics and for multilocus sequence typing (MLST).

**Results:**

The results showed that the 167 isolates showed the highest resistance to ampicillin (AMP, 87.4%), sulfamethoxazole (SF, 87.4%), compound sulfamethoxazole (SXT, 81.4%), nalidixic acid (NAL, 80.2%), and amoxicillin/clavulanic acid (A/C, 77.8%). All the strains were sensitive to meropenem (MEM), and 91.0% of the isolates were MDR strains. We screened a total of 45 ARGs, with the highest detection rate observed for the tetracycline (TET) resistance gene *tet* (A) (81.4%). A total of 21 types of plasmid replicons were detected in *Salmonella*, of which IncX1 was the most common (74.3%), and 62.9% of the isolates carried a class 1 integron. In addition, a total of 11 different serotypes were detected, with *S. enteritidis* as the predominant serovar., followed by *S. infantis* and *S.* Newport. Twelve different sequence types (STs) were detected, among which ST11 was the main type. There was a strong correspondence between serotypes and STs. We also found that *S. Indiana* and *S.* Kentucky had extremely high rates of resistance to ciprofloxacin (CIP) and third-generation cephalosporins. System-wide genome analysis showed the occurrence of long-distance transmission across fields.

**Conclusion:**

In conclusion, the detection of multidrug resistance and isolates carrying multidrug resistance genes is the main problem, and emergency strategies should be implemented to address this issue.

## Introduction

*Salmonella* is a common foodborne pathogen worldwide that is widely distributed in the environment and global food chain, posing a serious threat to food safety and public health. In 2019, the European Union reported that salmonellosis is the second most common human gastrointestinal infection after *Campylobacter* infection and is a significant cause of foodborne outbreaks in the EU, with 87,923 confirmed cases of human salmonellosis reported (1). To date, over 2,600 serovars of *Salmonella enterica* have been identified (2). In Europe, the vast majority (72.4%) of foodborne *Salmonella* outbreaks are caused by *S. enteritidis* (1). In China, 70% ~ 80% of foodborne bacterial outbreaks can be attributed to *Salmonella* infection, while *S. enteritidis* and *S. typhimurium* are the most common serotypes associated with human intestinal infections (3). In recent years, some new serotypes, such as *S.* Telkebier, *S.* Uzaramo, and *S.* Changwanni, have been gradually discovered in China (4–6).

Salmonellosis is usually associated with the ingestion of *Salmonella*-contaminated animal-derived foods, particularly chicken and poultry products, which are the most common source of transmission of *Salmonella* to humans (7). Many antibiotics are currently used in food animal production to promote growth and to prevent (prophylactic), treat (therapeutic), and control (metaphylactic) salmonellosis, but a serious problem associated with antibiotic use is the development of antibiotic resistance by pathogens. The level and degree of resistance are constantly changing worldwide and are affected by human and animal antimicrobial drug use practices and geographical differences (8). The indiscriminate use of antibiotics in animal husbandry has been identified as the driving factor for the development of multidrug-resistant (MDR) strains, and the resistance can be transmitted to humans through the food chain (9). For example, drug-resistant bacteria have been identified from various environmental samples, farms, and retail meat products (10–14). Over time, this will reduce the effectiveness of antibiotics and eventually lead to treatment failure. Therefore, the use of “crucial antibacterial drugs” such as fluoroquinolones and third-generation cephalosporins to treat *Salmonella* is classified as the highest priority, and these are the most important antibacterial drugs in human medicine, with colistin also being seen as the “last line of defense” for treating severe infections caused by MDR gram-negative pathogens (15).

Resistance can occur via point mutations in the bacterial genome or horizontal transfer through genetic elements carrying antibiotic resistance genes (ARGs) (16). The most effective way by which ARGs are transferred between microorganisms is horizontally transfer via movable genetic elements, which can be realized by the inclusion of one or more integrons, transposons, and plasmids that harbor an ARG (17, 18). This horizontal transfer ability increases the risk of treatment failure for clinical infections caused by S*almonella*. Therefore, it is particularly important to monitor the resistance of *Salmonella* to these antibiotics. Whole-genome sequencing (WGS), a molecular method for characterizing organisms, has been proven to be a fast, specific, cost-effective monitoring method compared to previous methods (19), and more importantly, WGS can be used to detect and predict emerging threats that may lead to severe human and animal infections in the early stages (20).

Shandong Province is the largest poultry production province in China, accounting for 30.0% of China’s total production (21). In China, especially in Shandong province, several recurrent outbreaks of avian salmonellosis have been reported during the last decade where the precise causal agent remains unknown. Previous studies on the prevalence and drug resistance of *Salmonella* in broiler chickens in Shandong Province are limited to a certain region or link, without large-scale horizontal research (22–24). This study characterized the relevant isolates of broiler chickens, and investigated the phenotypic and genotypic diversity of *Salmonella* from different sources and their genetic relationships by conducting drug sensitivity tests and WGS analysis on broiler chickens and their products in Shandong Province. Our findings contribute to assessing public health risks and provide insights into preventing *Salmonella* contamination and drug resistance.

## Materials and methods

### Sample collection

A total of 947 samples were randomly collected from 15 broiler farms (*n* = 618), 2 broiler slaughterhouses (*n* = 293), and 3 markets (*n* = 36) in Shandong Province, including cloacal swabs, environmental swabs (segmentation tools, water, containers), and carcass swabs. The sample set included 8 major chicken-producing cities in Shandong province. The number of cloacal samples collected at each farm ranged from 30 to 86, carcass samples collected at each slaughterhouse ranged from 73 to 125, environment samples collected at each slaughterhouse ranged from 35 to 60, and 12 chicken product samples were collected from each market. All samples were collected using eSwabs with 1 mL of Liquid Amies Medium (Copan Brescia, Italy). They were stored at 4°C in a refrigerator and transferred to the laboratory for processing within 24 h. The distribution information of the strains is shown in Supplementary Table S1.

### Isolation and identification of bacteria

The isolation of *Salmonella* was performed according to methods described previously (25). Briefly, fecal or cloacal swab samples were preenriched in 10 mL of buffered peptone water (BPW; Landbridge, Beijing, China). Following the initial preenrichment in BPW, 0.1 mL of the preenriched sample was added to 10 mL of selenite cystine broth (Landbridge, Beijing, China) and incubated at 37°C for 12 to 18 h. Final colony isolation was performed on xylose-lysine-tergitol-4 agar (BD Biosciences, United States). The positive *Salmonella* isolates were further identified by matrix-assisted laser desorption ionization–time of flight mass spectrometry (MALDI-TOF MS) (Bruker MALDI Biotyper System, Germany).

### Antimicrobial susceptibility testing

The minimum inhibitory concentrations (MICs) of *Salmonella* isolates were determined using the broth microdilution method, which employed the following 20 antimicrobial agents: ampicillin (AMP), amoxicillin/clavulanic acid (A/C), gentamicin (GEM), tetracycline (TET), ceftazidime (CAZ), colistin (CL) meropenem (MEM), sulfisoxazole (SF), spectinomycin (SPE), enrofloxacin (ENR), ofloxacin (OFX), amikacin (AMK), doxycycline (DOX), nalidixic acid (NAL), compound sulfamethoxazole (SXT), ceftiofur (CEF), ciprofloxacin (CIP), kanamycin (KAN), chloramphenicol (CHL), and florfenicol (FLO). *E. coli* ATCC 25922 was used as the control strain following the CLSI guidelines (26). The resistant breakpoints used were as follows: ≥32 mg/L for AMP, ≥32/16 mg/L for A/C, ≥8 mg/L for GEN, ≥16 mg/L for TET, ≥16 mg/L for CAZ, ≥4 mg/L for CL, ≥4 mg/L for MEM, ≥512 mg/L for SF, ≥2 mg/L for ENR, ≥8 mg/L for OFX, ≥16 mg/L for AMK, ≥16 mg/L for DOX, ≥32 mg/L for NAL, ≥4/76 mg/L for SXT, ≥4 mg/L for CIP, ≥64 mg/L for KAN, and ≥ 32 mg/L for CHL (CLSI M100-ED33). While the resistance breakpoint used for SPE (≥128 mg/L) was followed by the clinical breakpoint for *Pasteurella multocida-bovine*, CEF (≥8 mg/L) and FLO (≥16 mg/L) were followed by the clinical breakpoint for Enterobacterales-swine (CLSI VET01S ED6). Resistance to three or more classes of drugs was considered multiple drug resistance.

### Whole-genome sequencing analysis

The TIANamp Bacteria DNA Kit (Tiangen Biotech, Beijing, China) was used according to the manufacturer’s recommendations to extract the genomic DNA of 70 selected *Salmonella* strains (the selection of the 70 *Salmonella* strains was based on drug sensitivity, different regional sources, and different production processes). Subsequently, bacterial DNA quality testing and construction of the *Salmonella* DNA library were carried out, and WGS was performed using the Illumina NovaSeq 6,000 platform (Sinobiocore, Beijing, China). Online tools were used to submit complete nucleotide sequences to the web server of the Genome Epidemiology Center^1^ for plasmid typing, ARG identification, and multilocus sequence typing (MLST). In addition, a type of integron was obtained through gene annotation on the Rast website.^2^ The core gene was defined as a gene that was present in ≥99% of the genomes. All core genes were used to construct phylogenetic trees using Fasttree v.2.1.11. Heatmaps of the clustering of ARGs and serotypes were created by using TBtools v.1.116.

### Data analysis

The analysis of the obtained results and the generation of figures were performed using the GraphPad Prism (GraphPad, San Diego, CA, United States) version 7.03, and Student’s *t*-test with Welch’s correction were used in this study.

## Results

### Antibiotic resistance and MDR profiles

**Figure 1:**
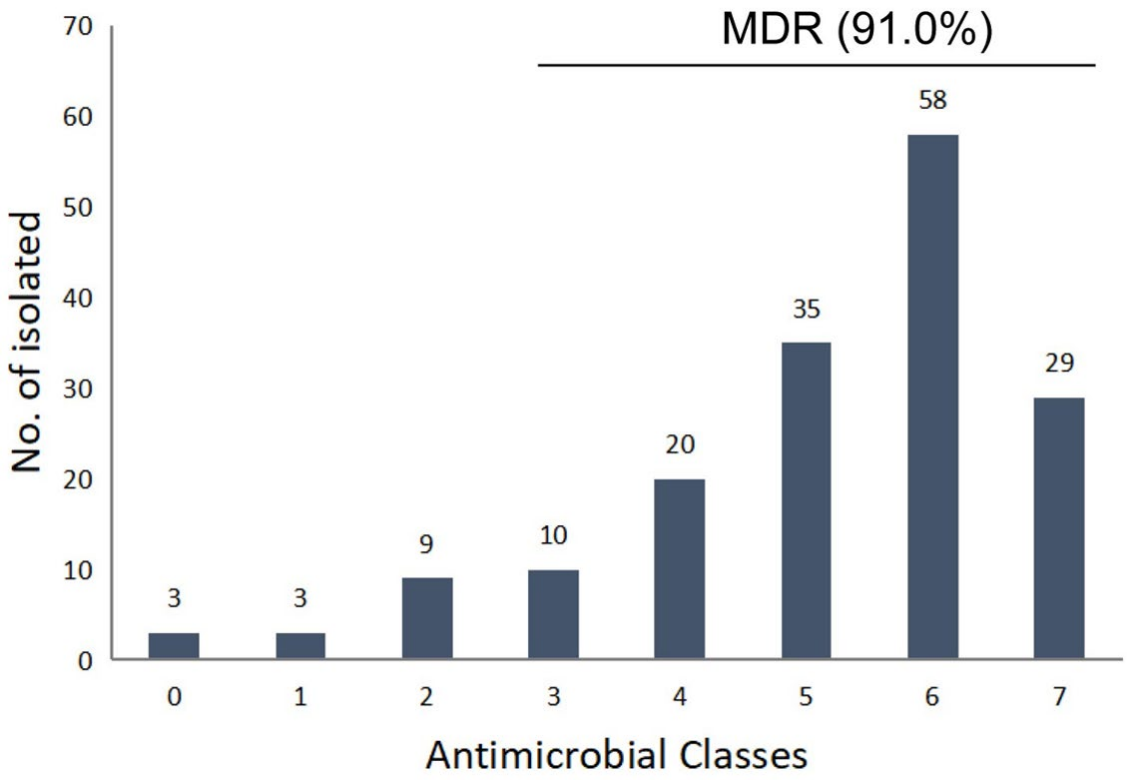
Multidrug resistance profile pattern of 167 *Salmonella* strains.

Among the 947 samples, a total of 167 *Salmonella* strains were identified, and the total contamination rate of broilers and products was 17.6%, with a positivity rate of 32.8% in slaughterhouses, which is significantly higher than poultry farms (11.2%) and market samples (5.6%), respectively (*p* < 0.05). The antimicrobial susceptibility testing results showed that the obtained *Salmonella* isolates had the highest resistance (˃80%) to AMP (87.4%), SF (87.4%), SXT (81.4%), NAL (80.2%), high resistance (50–80%) to A/C (77.8%), TET (66.5%), DOX (65.3%), CL (62.9%), FLO (53.2%), and CHL (51.5%), and moderate resistance (30–50%) to KAN (47.9%), SPE (47.9%), CEF (44.9%), OFX (33.5%) (Table 1). A total of 26.3 and 21.0% of the strains showed resistance to third-generation cephalosporins and CIP, respectively (Table 1). 94.4% of CIP resistant strains are also resistant to NAL and OFX, and 61.1% of CIP resistant strains are also resistant to ENR. Most of the drugs had high MIC_50_ and MIC_90_ levels, especially AMP, SF and NAL, the MIC_50_ and MIC_90_ of which were higher than the highest tested concentration (>128 mg/L, >512 mg/L, and >128 mg/L, respectively). The isolates showed lower resistance to AMK (5.4%), and all the strains showed sensitivity to MEM. Out of the 167 isolates, 164 (98.2%) were resistant to at least one antimicrobial agent, while 3 (1.8%) were sensitive to all the tested antimicrobial agents. A total of 152 isolated strains (91.0%) showed multidrug resistance, with 5 and 6 strains accounting for the largest proportion, accounting for 21.0 and 34.7% of the total bacterial count, respectively (Figure 1). The isolates from poultry farms showed the highest resistance to AMP, NAL, SF, SXT, and FLO, with multiple resistant strains accounting for 91.3% of the total number of isolates from farms; the strains isolated from slaughterhouses showed the highest resistance to SF, AMP, SXT, A/C, NAL, and CL, with multiple resistant strains accounting for 90.6% of the total number of strains isolated from slaughterhouses; the strains sourced from markets are all multidrug-resistant strains (Supplementary Table S2).

**Table 1:** Distributions of the MICs of 167 *Salmonella* isolates against 20 antimicrobial agents.

Antibiotic category	Antibiotics	MIC value (mg/L)		No. of resistant isolates (%)
		MIC_50_	MIC_90_	
β-Lactams	AMP	>128	>128	146 (87.4)
	A/C	64/32	>128/64	130 (77.8)
	CAZ	1	64	44 (26.3)
	CEF	1	>128	75 (44.9)
	MEM	≤0.0625	0.125	0
Aminoglycosides	GEM	1	64	60 (35.9)
	SPE	64	>256	80 (47.9)
	AMK	4	8	9 (5.4)
	KAN	32	>512	80 (47.9)
Tetracyclines	TET	64	>128	111 (66.5)
	DOX	32	64	109 (65.3)
Sulfonamides	SF	>512	>512	146 (87.4)
	SXT	32/608	>32/608	136 (81.4)
Polypeptides	CL	4	8	105 (62.9)
Quinolones	NAL	>128	>128	134 (80.2)
	OFX	0.5	64	56 (33.5)
	ENR	0.5	8	25 (15.0)
	CIP	≤0.25	2	35 (21.0)
Amide alcohols	CHL	128	256	86 (51.5)
	FLO	128	>128	89 (53.2)

AMP, ampicillin; A/C, amoxicillin + clavulanic acid; CAZ, ceftazidime; CEF, cefotiofur; MEM, meropenem; GEM, gentamicin; SPE, spectinomycin; AMK, amikacin; KAN, kanamycin; TET, tetracycline; DOX, doxycycline; SF, sulfisoxazole; SXT, sulfamethoxazole + trimethoprim; CL, colistin; NAL, nalidixic acid; OFX, ofloxacin; ENR, enrofloxacin; CIP, ciprofloxacin; CHL, chloramphenicol; FLO, florfenicol. MIC_50_ = MIC value at which growth was inhibited in 50% of isolates; MIC_90_ = MIC value at which growth was inhibited in 90% of isolates.

### Antimicrobial resistance genes and serotypes

**Figure 2:**
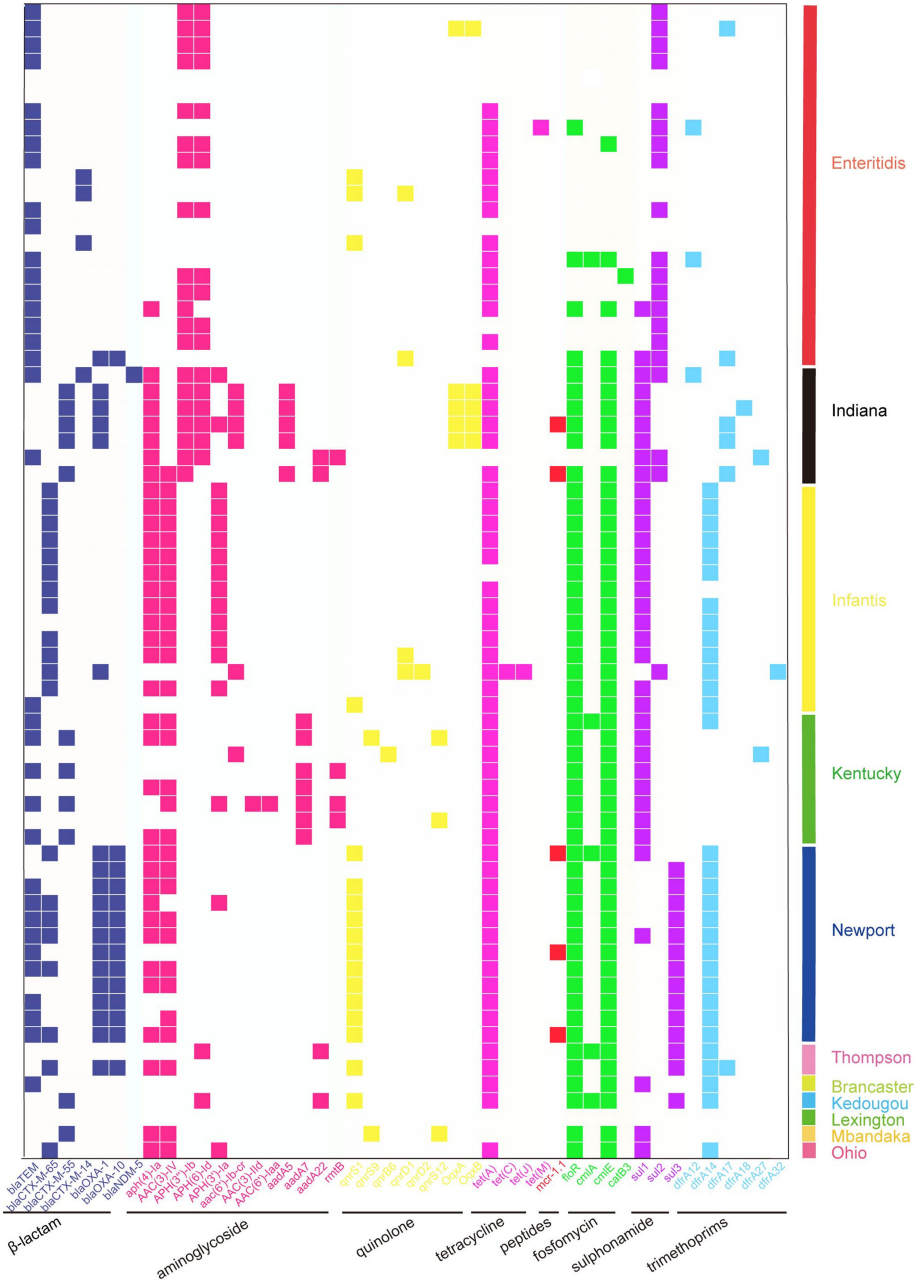
Heatmap showing the ARG profiles in this work. Different groups of ARGs are color-coded. Distribution of ARGs among different serotypes of *Salmonella*.

A total of 45 ARGs were screened from the 70 sequenced strains, and 81.4% of the isolates were found to carry the *tet* (A) gene, conferring TET resistance. The carriage rates of the *floR* and *cmlE* genes were both 71.4%, which endowed the strains with resistance to phenolic drugs. The detection rate of β-lactam resistance genes was the highest for *bla*_TEM_ (50.0%). The detection rate of aminoglycoside resistance genes was the highest in the *aph (4) - Ia group* (51.4%); the detection rate of quinolone resistance genes was highest for *qnrS1* (31.4%). We also found that 7.1% of the strains carried *mcr-1* colistin resistance genes, which is a particularly noteworthy finding (Supplementary Table S2). A total of 11 different serotypes were identified by sequencing, with *S. enteritidis* being the main serotype (31.4%), followed by *S. infantis* (20.0%), *S.* Newport (17.1%), *S.* Kentucky (11.4%), *S. Indiana* (10.0%), and *S.* Thompson (2.9%). In addition, *S.* Kedougou, *S.* Mbandaka, *S.* Ohio, and *S.* Lexington were also detected. The carriage of ARGs varied among the different serotype isolates, and compared to other serotypes, *S. Indiana* carried the most ARGs (Figure 2).

### Plasmid typing and class 1 integrons

**Figure 3:**
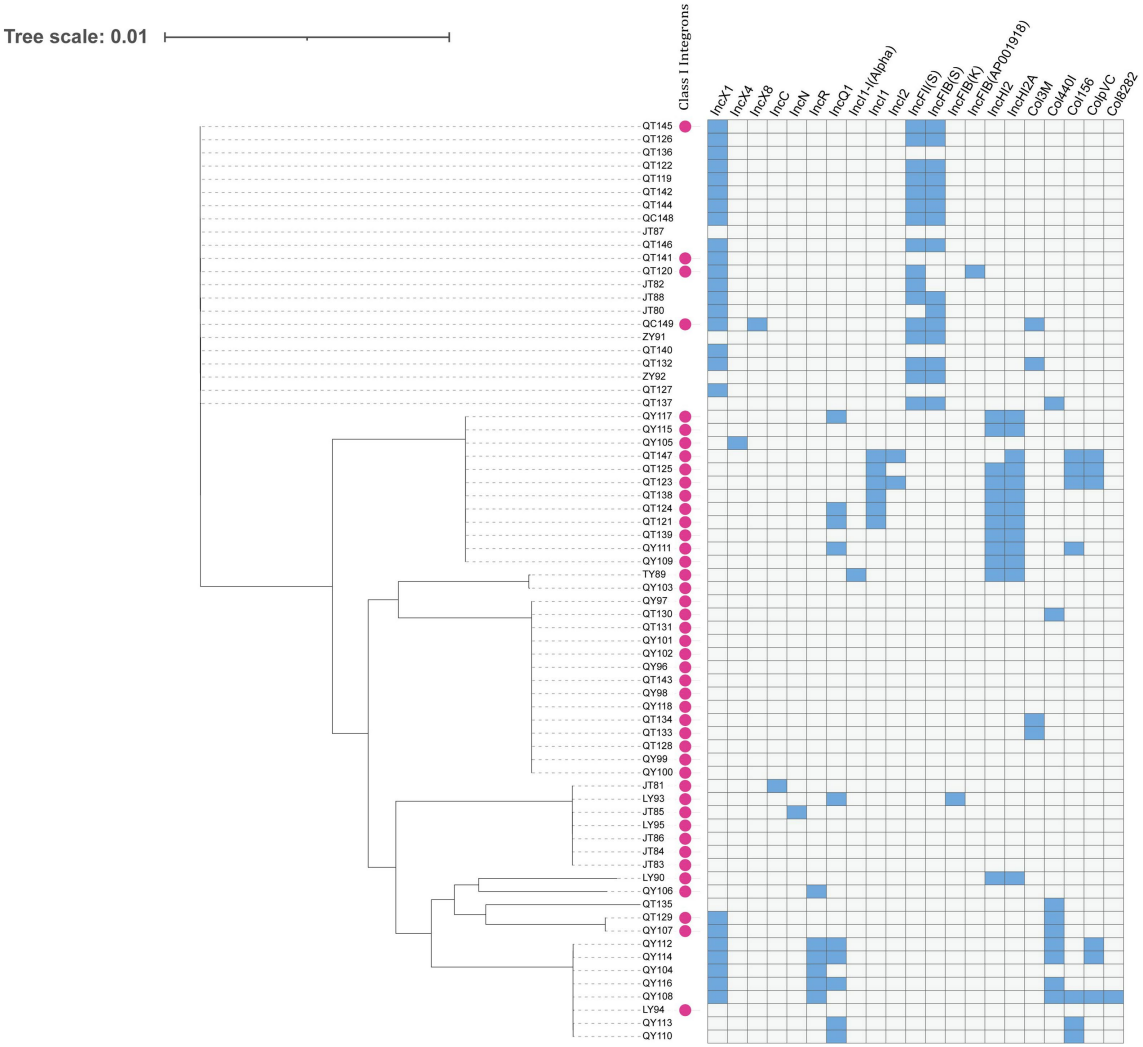
Dendrogram of a hierarchical clustering heatmap of the 70 isolates, 21 plasmid replicons, and class 1 integrons. Blue cells represent the presence of plasmid; gray cells represent the absence of plasmid. Class 1 integrons are represented by purple circles.

A total of 21 replicon types were detected from all the sequenced strains. Among the plasmid replicon types, IncX1, IncFII (S), IncFIB (S), IncHI2A, IncHI2, and IncQ1 were the most common, and the rest included IncX4, IncX8, IncC, IncN, IncI1-I (Alpha), IncFIB (K), Col8282, IncI2, IncFIB (AP001918), Col3M, IncR, IncI1, ColpVC, Col156, and Col440I. The total detection rate for class I integrons was 62.9%. All *Salmonella* strains carrying integrons were MDR strains (Supplementary Table S2). Most of the strains carried class I integrons and plasmid replicons (Figure 3).

### MLST and phylogenetic relationships

**Figure 4:**
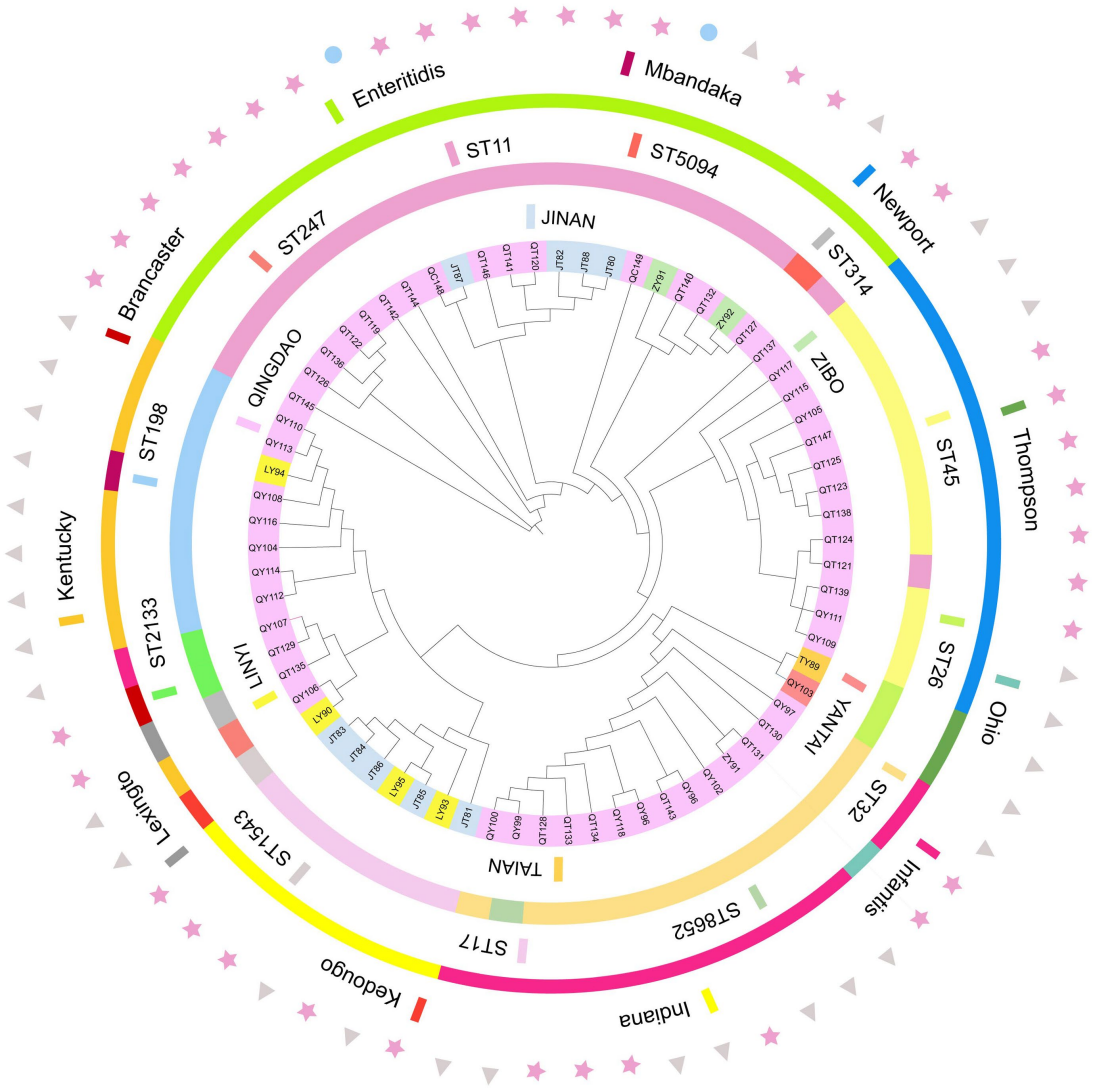
Phylogenetic relationships of 70 strains of bacteria. The tree was created using the annotated iTOL interactive user interface (https://itol.embl.de). The circles, from inside to outside, indicate the regional sources of strains (circle 1), the ST of the strain (circle 2), and the serotype type of the strain (circle 3); triangles represents the farms, stars represents the slaughterhouses, and circles represents the markets (circle 4).

A total of 12 sequence types (STs) were identified from the 70 sequenced strains, with ST11 being the main type, followed by ST32 and ST45. Other sequence types included ST198, ST17, ST26, ST2133, ST247, ST314, ST1543, ST5094, and ST8652. In this study, 95.5% of the *S. enteritidis* strains were ST11, 91.7% of the *S.* Newport strains were ST45, 92.3% of the *S. infantis* strains were ST32, 100% of the *S. Indiana* strains were ST17, and the *S.* Kentucky strains were mainly ST198, which indicated that serotype and ST have a very high coincidence rate. There were 10 STs in the broiler farms, with ST198 being the main type. There were 7 STs in the slaughterhouses, with ST11 being the main type. The ST of the market samples was ST11. The *Salmonella* phylogenetic tree shows that a total of 10 main branches were formed. Strains from the same region were closer in evolutionary branches and shared close genetic relationships; there were also close genetic relationships between the same serotype and ST. Among the samples, market samples were located in two separate evolutionary branches, indicating that there were other sources of contamination besides farms and slaughterhouses (Figure 4).

## Discussion

In this study, the positivity rate in poultry farms in Shandong Province was 11.2%, slightly higher than the positivity rate in broiler farms in Southeast Asia and the Zhejiang and Fujian regions (27, 28). *Salmonella* in slaughterhouses has been detected at various stages of slaughter, indicating that it can spread along the slaughter line. The *Salmonella* detection rate in the market samples was consistent with previous research results (29).

Due to the excessive use of antibiotics and horizontal transmission of ARGs in animal husbandry in the past decades, *Salmonella* antibiotic resistance has become a global threat (30). The antibiotics selected in this study are the most commonly used classes in the poultry production chain in Shandong province, long-term use of these antibiotics may lead to antibiotic resistance issues (31). Among the 167 strains of *Salmonella* in this study, 152 (91.0%) were resistant to more than three drugs and widely resistant to conventional antibiotics. The MIC_50_ and MIC_90_ can reflect the drug resistance of *Salmonella* as a whole. The results showed that the resistance rate to sulfonamides, AMP, and NAL was the highest, and the resistance to A/C and TET was also generally high. The MIC_50_ and MIC_90_ of these antibiotics also far exceeded the breakpoint for drug resistance, which may be because these are the antibiotics most commonly used to treat several infectious diseases in poultry. The resistance to third-generation cephalosporins was moderate. The resistance rate for the quinolone drug NAL reached 80.2% (MIC_50_ > 128 mg/L, MIC_90_ > 128 mg/L), and this high resistance rate has also been found elsewhere in China and other regions (21, 32–34), especially for the combinations of trimethoprim and sulfonamides. This situation may be due to the widespread use of quinolone drugs in livestock and poultry breeding feed in China, leading to selective pressure on bacteria (30). In the 70 strains sequenced, 45 ARGs were detected, and the isolated strains carried different resistance genes, which is probably related to the complexity of the strain resistance phenotypes. The resistance genes of most of the strains were consistent with their phenotypes, while some strains had certain resistance phenotypes but did not carry the corresponding ARGs, which may be related to unknown resistance mechanisms or the nonspecific functioning of multiple redundant efflux pump-like genes. Similarly, the presence of resistance genes does not necessarily lead to phenotypic resistance, and sometimes isolates show antibiotic resistance without harboring known resistance genes (35). In our study, *bla*_TEM_ was the most common resistance gene. The detection rate of the aminoglycoside resistance gene *aph (4) - Ia* was the highest, and a small number of strains harbored the *aac (6’) - Ib-cr* gene. The *aac (6’) - Ib-cr* gene is a key mediator of bacterial resistance to CIP (36). In addition, multiple quinolone resistance genes were detected, including *qnrS1, OqxA, OqxB, qnrS12, qnrD1, qnrS9, qnrB6,* and *qnrD2*. The high prevalence of plasmid-mediated quinolone resistance (PMQR) genes highlights the importance of cautious use of fluoroquinolone drugs to minimize fluoroquinolone resistance.

Serotype analysis showed significant diversity, with *S. enteritidis* present in most samples, indicating that it has an absolute advantage, which is consistent with the results of other studies in China (37, 38). *S. Indiana* may have higher resistance potential, as it carried more ARGs. More importantly, *S. Indiana* was 100% resistant to CIP and third-generation cephalosporins, and one of the *S. Indiana* strains also carried the *bla*_NDM-5_ resistance gene. In addition, *S.* Kentucky was also 100% resistant to CIP and had high resistance to third-generation cephalosporins, which was similar to previous research results (39). The extended-spectrum beta-lactamase (ESBL) gene is the most important determinant of third-generation cephalosporin resistance in *Salmonella*, and *bla*_CTX-M-55_ is the dominant ESBL gene. In our study, this ARG was mainly detected in the Indiana and Kentucky serotypes, and the *bla*_CTX-M-55_ gene was also mainly detected in *S.* Kentucky (29). CIP and cephalosporins are the drugs most commonly used for treating salmonellosis in humans. The dual resistance to CIP and third-generation cephalosporins poses an enormous threat to human health, as treatment failure may have serious consequences (40). Therefore, *S. Indiana* and *S.* Kentucky may have a higher risk of drug resistance. We also discovered a certain number of *S.* Newport serotypes, which have caused several outbreaks in the United States, infecting millions of people annually (41), and are often detected in poultry chains in Brazil (42). There have been reports of this serotype in other provinces of China, but it has not caused large-scale outbreaks in China. Resistance to CL is believed to be mainly caused by the *mcr-1* gene located on transferable plasmids, which was first discovered in China from animals, food, and humans (43). In our study, the *S.* Newport isolates were 100% resistant to CL, with three strains carrying the *mcr-1* gene (44). *S.* Newport with *mcr-1* positivity was also found among the chicken isolates. One of the three *mcr-1*-positive strains carried the IncX4 plasmid, while the other two *mcr-1*-positive strains carried the IncI2 plasmid, and all three strains carried class 1 integrons. Some studies have shown that the main types of replicons carrying the *mcr-1* plasmid are IncI2, IncX4, IncHI2 and IncP, which is consistent with our research. In other studies, the *mcr-1* gene was also found on the IncX4 and IncI2 plasmids (45). In addition, studies have shown that *mcr-*positive IncX4 plasmids may spread between different bacterial species, from animals to humans or from humans to animals (46). Plasmids are considered the main mobile elements that determine the horizontal transfer of genes (47). In our study, a total of 21 different plasmids were detected, among which IncX1 and IncFII (S) were dominant plasmids. These plasmids were distributed in different regions, breeding stages, and serotypes, indicating that these plasmids can be widely spread among different serotypes and regions. Integrons are mobile genetic elements encoding bacterial genes related to antibiotic resistance that can be transmitted between microorganisms. The most common integron in MDR *Salmonella* is the class 1 integron (48). All the *S. Indiana*, *S. infantis*, *S.* Newport, and *S.* Thompson strains that we isolated carried class 1 integrons, while only three strains of *S. enteritidis* carried class 1 integrons. We also found that some ARGs, plasmid replicons and class 1 integrons existed in the same strain simultaneously, which may be highly conducive to the horizontal transmission of drug resistance among strains.

We found a strong correspondence between serotypes and STs, and this phenomenon has also been observed in other studies (31). Due to these correlations, it is possible to predict *Salmonella* serotypes to some extent. The phylogenetic tree results showed that strains with the same sequence type readily clustered together, confirming the genetic homology of isolates from the same serotype/ST. The strains isolated from the same farm clustered together, indicating that the strains from the same farm shared a common ancestor, originating from the same clone, and that clonal transmission occurred in the farm, which was likely caused by a lack of disinfection measures in the farm, which allowed pathogens to spread in the field and persist in the farm, resulting in vertical transmission (49). In addition, there were very close genetic relationships among the isolates from four farms in the same region, and these farms may have harbored the same cloned strains. Strains ZY91, QT140, QT132, ZY92, and QT127 clustered together, but they were derived from different regions, isolation stages, and sample sources. This indicates that clones of the same strain may spread over long distances through broiler trading or food production chains, such as those of chicken. As the last link in the entire broiler production chain, broiler products are closely related to human life. Two chicken samples from markets were located in two separate branches, indicating that there were other sources of transmission besides farms and slaughterhouses, during product transportation. The strains isolated from markets were of the serotype *S. enteritidis*. Which is a major cause of foodborne disease outbreaks (50). *Salmonella* surveillance data show that since the 1970s, the incidence rate of *S. enteritidis* in food has been on the rise worldwide (51), and this global growth is related to chicken products, such as poultry meat and eggs (52). There is an urgent need to draw attention to this issue and adopt appropriate measures in animal husbandry and the cooking of food to reduce the occurrence of *Salmonella* in poultry and poultry products. Monitoring plans should be implemented at all stages of poultry production, including during breeding of chicken flocks, in hatcheries, in broiler flocks, in slaughterhouses and at sale sites, which could significantly reduce *Salmonella* contamination of broilers and broiler products. More importantly, the two isolates from the market were both MDR strains, and they carried a variety of ARGs and plasmids, posing a high risk of antibiotic resistance transfer. The emergence and spread of drug-resistant *Salmonella* from food animals or retail meat have become severe health hazards worldwide, posing a serious threat to public health.

## Conclusion

This study found that there was a considerable amount of *Salmonella* contamination in broiler farms, slaughterhouses, and processing and retail sites in Shandong, with a variety of serotypes detected. In addition, some *Salmonella* strains isolated in this study were MDR strains that endanger public health and have the potential to spread horizontally, posing a serious risk to food safety. Therefore, it is necessary to implement stricter medication management systems to minimize the risk of further promoting the spread of drug-resistant strains of these dangerous bacteria.

## Data availability statement

The data presented in the study are deposited in the NCBI repository, accession number PRJNA1013903.

## Ethics statement

The animal studies were approved by Qingdao Agricultural University. The studies were conducted in accordance with the local legislation and institutional requirements. Written informed consent was obtained from the owners for the participation of their animals in this study.

## Author contributions

LZ: Data curation, Formal analysis, Methodology, Writing – original draft. GL: Conceptualization, Supervision, Writing – review & editing. WT: Conceptualization, Writing – review & editing, Project administration. XS: Conceptualization, Project administration, Writing – review & editing. XZ: Conceptualization, Project administration, Writing – review & editing. CW: Writing – review & editing, Data curation. YL: Writing – review & editing, Funding acquisition. MZ: Funding acquisition, Writing – review & editing.

## Footnotes

^1^ https://cge.cbs.dtu.dk/services/

^2^ https://rast.nmpdr.org/
