# Supplementary material for: Antimicrobial resistance and genomic characteristics of Salmonella from broilers in Shandong Province
Source: Front Vet Sci. 2023 Nov 23;10:1292401. doi: 10.3389/fvets.2023.1292401 (PMC10701519; doi:10.3389/fvets.2023.1292401)
Supplement: Supplementary file 1 [file Data_Sheet_1.docx]

Supplementary Material

# Table S1. Pollution of *Salmonella* in Broilers in Shandong Province

| Soures | Area | Type | Percentage of *Salmonella* isolates (n) |
| --- | --- | --- | --- |
| Farm 1 | Taian | Cloaca swab | 3.3%（1/30） |
| Farm 2 | Linyi | Cloaca swab | 40.0%（12/30） |
| Farm 3 | Linyi | Cloaca swab | 26.7%（8/30） |
| Farm 4 | Zibo | Cloaca swab | 23.3%（7/30） |
| Farm 5 | Qingdao | Cloaca swab | 50.0%（15/30） |
| Farm 6 | Yantai | Cloaca swab | 13.3%（4/30） |
| Farm 7 | Zaozhuang | Cloaca swab | 0.0%（0/30） |
| Farm 8 | Zaozhuang | Cloaca swab | 0.0%（0/30） |
| Farm 9 | Zaozhuang | Cloaca swab | 0.0%（0/30） |
| Farm 10 | Jining | Cloaca swab | 0.0%（0/30） |
| Farm 11 | Jining | Cloaca swab | 0.0%（0/30） |
| Farm 12 | Weifang | Cloaca swab | 0.0%（0/30） |
| Farm 13 | Qingdao | Cloaca swab | 7.0%（6/86） |
| Farm 14 | Qingdao | Cloaca swab | 11.6%（10/86） |
| Farm 15 | Qingdao | Cloaca swab | 7.0%（6/86） |
| Slaughterhouse 1 | Jinan | Carcass | 23.3%（17/73） |
| Slaughterhouse 1 | Jinan | Environment | 28.6%（10/35） |
| Slaughterhouse 2 | Qingdao | Carcass | 36.0%（43/125） |
| Slaughterhouse 2 | Qingdao | Environment | 43.3%（26/60） |
| Market 1 | Qingdao | Chicken products | 8.3%（1/12） |
| Market 2 | Qingdao | Chicken products | 8.3%（1/12） |
| Market 3 | Qingdao | Chicken products | 0.0%（0/12） |
| Total |  |  | 17.6%（167/947） |

# Table S2. Serovar, resistance phenotype, incidence of class 1 integron, and resistance genes in *Salmonellla* isolated from broilers in Shandong Province

| Strain | Serovar | Antimicrobial resistance pattern | ARG | Integrons |
| --- | --- | --- | --- | --- |
| JY80 | Enteritidis | AMP-A/C-GEM-TET-SF-NAL | blaTEM, aph(6)-Id, aph(3'')-Ib, sul2 | - |
| JT82 | Enteritidis | AMP-A/C-CL-SF-NAL-SXT | blaTEM, aph(3'')-Ib, aph(6)-Id, sul2, OqxA, OqxB | - |
| JT87 | Enteritidis | AMP-A/C-CL-SF-NAL-SXT | blaTEM, aph(6)-Id, aph(3'')-Ib, sul2 | - |
| JT88 | Enteritidis | AMP-A/C-SF-OFX-NAL-SXT | blaTEM, aph(6)-Id, aph(3'')-Ib, sul2 | - |
| ZY91 | Enteritidis | CL-NAL | - | - |
| ZY92 | Enteritidis | AMP-A/C-GEM-TET-CAZ-CL-SF-SPE-NAL-SXT-CEF-FLO | - | - |
| QT119 | Enteritidis | AMP-A/C-TET-CL-SF-DOX-NAL-SXT-FLO | blaTEM, aph(3'')-Ib, aph(6)-Id, tet(A), sul2 | - |
| QT120 | Enteritidis | AMP-A/C-TET-CL-SF-SPE-DOX-NAL-SXT-CHL-FLO | blaTEM, aadA1, aadA2, tet(A), tet(M), sul2, dfrA12, floR, cmlE | + |
| QT122 | Enteritidis | AMP-A/C-TET-CL-SF-DOX-NAL-SXT-KAN-FLO | blaTEM, aph(3'')-Ib, aph(6)-Id, tet(A), sul2 | - |
| QT126 | Enteritidis | AMP-A/C-TET-CL-SF-DOX-NAL-SXT-KAN-CHL-FLO | blaTEM, aph(3'')-Ib, aph(6)-Id, tet(A), sul2 | - |
| QT127 | Enteritidis | AMP-A/C-TET-CL-SF-OFX-DOX-NAL-SXT-CEF-KAN-CHL-CIP-FLO | blaCTX-M-14, tet(A), qnrS1 | - |
| QT132 | Enteritidis | AMP-A/C-TET-CAZ-CL-SF-SPE-OFX-DOX-NAL-SXT-CEF | blaCTX-M-14, tet(A), qnrS1, qnrD1 | - |
| QT136 | Enteritidis | AMP-A/C-TET-CAZ-CL-SF-DOX-NAL-SXT | blaTEM, aph(3'')-Ib, aph(6)-Id, tet(A), sul2 | - |
| QT137 | Enteritidis | AMP-A/C-GEM-CL-SF-NAL-KAN | blaTEM, aph(3')-IIa | - |
| QT140 | Enteritidis | AMP-A/C-TET-CL-SF-SPE-OFX-DOX-NAL-SXT-CEF-CHL | blaCTX-M-14, tet(A), qnrS1, floR, cmlA, cmlE | - |
| QT141 | Enteritidis | AMP-A/C-TET-CL-SF-SPE-DOX-NAL-SXT-CHL-FLO | blaTEM, aadA1, aadA2, tet(A), sul2, dfrA12, floR, cmlA, cmlE | + |
| QT142 | Enteritidis | AMP-TET-SF-DOX-NAL | blaTEM, aph(6)-Id, aph(3'')-Ib, tet(A), sul2, catB3 | - |
| QT144 | Enteritidis | AMP-A/C-TET-CL-SF-DOX-NAL-SXT | blaTEM, aph(6)-Id, aph(3'')-Ib, tet(A), sul2 | - |
| QT145 | Enteritidis | AMP-A/C-TET-CL-SF-DOX-NAL | blaCMY-2, blaTEM, , aac(6')-Il, aph(4)-Ia, aph(3'')-Ib, tet(A), sul1, sul2, floR, cmlE | + |
| QT146 | Enteritidis | AMP-A/C-SF-NAL | blaTEM, aph(3'')-Ib, aph(6)-Id, sul2 | - |
| QC148 | Enteritidis | AMP-A/C-TET-SF-DOX-NAL | blaTEM, , aph(6)-Id, aph(3'')-Ib, tet(A), sul2 | - |
| QC149 | Enteritidis | AMP-A/C-CL-SPE-DOX-NAL-CEF-KAN-CHL-FLO | blaOXA-10, blaTEM, addA5, sul1, sul2, dfrA17, qnrD1, floR, cmlE | + |
| QY105 | Newport | AMP-A/C-GEM-TET-CL-SF-SPE-ENR-OFX-AMK-DOX-NAL-SXT-CEF-KAN-CHL-CIP-FLO | blaOXA-10, blaCTX-M-65, aph(4)-Ia, aac(3)-IV, tet(A), sul1, dfrA14, mcr-1.1, qnrS1, floR, cmlA, cmlE | + |
| QY109 | Newport | AMP-A/C-GEM-TET-CL-SF-ENR-OFX-DOX-SXT-KAN-CHL-FLO | blaOXA-10, aac(3)-IV, aph(4)-Ia, tet(A), sul3, dfrA14, qnrS1, floR, cmlE | + |
| QY111 | Newport | AMP-A/C-GEM-TET-CL-SF-SPE-DOX-SXT-KAN-CHL-FLO | blaOXA-10, blaTEM, aph(4)-Ia, aac(3)-IV, tet(A), sul3, dfrA14, qnrS1, floR, cmlE | + |
| QY115 | Newport | AMP-A/C-GEM-TET-CL-SF-SPE-DOX-SXT-CEF-KAN-CHL-FLO | blaTEM, blaOXA-10, blaCTX-M-65, aph(3')-Ia, aph(4)-Ia, tet(A), sul3, dfrA14, qnrS1, floR, cmlE | + |
| QY117 | Newport | AMP-A/C-GEM-TET-CL-SF-SPE-DOX-SXT-CEF-KAN-CHL-FLO | blaTEM, blaOXA-10, blaCTX-M-65, aph(4)-Ia, aac(3)-IV, tet(A), sul3, dfrA14, qnrS1, floR, cmlE | + |
| QT121 | Newport | AMP-A/C-GEM-TET-CAZ-CL-SF-SPE-DOX-SXT-CEF-KAN-CHL-FLO | blaOXA-10, blaCTX-M-65, blaTEM, aph(4)-Ia, aac(3)-IV, tet(A), sul1, sul3, dfrA14, qnrS1, floR, cmlE | + |
| QT123 | Newport | AMP-A/C-TET-CL-SF-SPE-DOX-SXT-CHL-FLO | blaTEM, blaOXA-10, tet(A), sul3, dfrA14, mcr-1.1, qnrS1, floR, cmlE | + |
| QT124 | Newport | AMP-A/C-GEM-TET-CAZ-CL-SF-SPE-DOX-SXT-CEF-KAN-CHL | blaTEM, blaOXA-10, blaCTX-M-65, aac(3)-IV, aph(4)-Ia, tet(A), sul3, dfrA14, qnrS1, floR, cmlE | + |
| QT125 | Newport | AMP-A/C-GEM-TET-CL-SF-SPE-DOX-SXT-KAN-CHL-FLO | blaOXA-10, aph(4)-Ia, aac(3)-IV, tet(A), sul3, dfrA14, qnrS1, floR, cmlE | + |
| QT138 | Newport | AMP-A/C-TET-CL-SF-DOX-NAL-SXT-CHL-FLO | blaTEM, blaOXA-10, tet(A), sul3, dfrA14, qnrS1, floR, cmlE | + |
| QT139 | Newport | AMP-A/C-GEM-TET-CL-SF-SPE-OFX-DOX-SXT-KAN-CHL-FLO | blaTEM, blaOXA-10, aac(3)-IV, tet(A), sul3, dfrA14, qnrS1, floR, cmlE | + |
| QT147 | Newport | AMP-A/C-GEM-TET-CL-SF-SPE-DOX-NAL-SXT-CEF-KAN-CHL-FLO | blaTEM, blaOXA-10, blaCTX-M-65, aph(4)-Ia, aac(3)-IV, tet(A), sul3, dfrA14, mcr-1, qnrS1, floR, cmlE | + |
| QY96 | Infantis | AMP-A/C-TET-SF-SPE-OFX-DOX-NAL-SXT-CEF-KAN-CHL-FLO | blaCTX-M-65, aph(4)-Ia, aac(3)-IV, aph(3')-Ia, tet(A), sul1, dfrA14, floR, cmlE | + |
| QY97 | Infantis | AMP-A/C-TET-CAZ-SF-SPE-OFX-DOX-NAL-SXT-CEF-KAN-CHL-CIP-FLO | blaCTX-M-65, aph(4)-Ia, aac(3)-IV, aph(3')-Ia, tet(A), sul1, dfrA14, floR, cmlE | + |
| QY98 | Infantis | AMP-A/C-GEM-TET-SF-SPE-OFX-DOX-NAL-SXT-CEF-KAN-CHL-FLO | blaCTX-M-65, aph(4)-Ia, aac(3)-IV, aph(3')-Ia, tet(A), sul1, dfrA14, floR, cmlE | + |
| QY99 | Infantis | AMP-A/C-TET-CAZ-SF-SPE-OFX-DOX-NAL-SXT-CEF-KAN-CHL-FLO | blaCTX-M-65, aph(4)-Ia, aac(3)-IV, aph(3')-Ia, tet(A), sul1, dfrA14, floR, cmlE | + |
| QY100 | Infantis | AMP-TET-SF-SPE-OFX-DOX-NAL-SXT-CEF-KAN-CHL-CIP-FLO | blaCTX-M-65, aph(4)-Ia, aac(3)-IV, aph(3')-Ia, tet(A), sul1, dfrA14, floR, cmlE | + |
| QY101 | Infantis | AMP-GEM-TET-SF-SPE-OFX-DOX-NAL-SXT-CEF-KAN-CHL-FLO | blaCTX-M-65, aac(3)-IV, aph(4)-Ia, aph(3')-Ia, sul1, dfrA14, floR, cmlE | + |
| QY102 | Infantis | AMP-TET-SF-SPE-OFX-DOX-NAL-CEF-KAN-CHL-FLO | blaCTX-M-65, aph(3')-Ia, aph(4)-Ia, aac(3)-IV, tet(A), sul1, floR, cmlE | + |
| QY107 | Infantis | AMP-A/C-TET-CL-SF-OFX-DOX-SXT-KAN-CHL-FLO | blaTEM-176, tet(A), sul1, dfrA14, qnrS1, floR, cmlE | + |
| QY118 | Infantis | AMP-A/C-GEM-TET-SF-SPE-DOX-NAL-SXT-CEF-KAN-CHL-FLO | blaCTX-M-65, aac(3)-IV, aph(4)-Ia, aph(3')-Ia, tet(A), sul1, dfrA14, floR, cmlE | + |
| QT128 | Infantis | AMP-A/C-TET-CAZ-CL-SF-SPE-DOX-NAL-SXT-CEF-KAN-CHL-FLO | blaCTX-M-65, aph(4)-Ia, aac(3)-IV, aph(3')-Ia, tet(A), sul1, dfrA14, floR, cmlE | + |
| QT130 | Infantis | AMP-A/C-CAZ-CL-SF-NAL-SXT-CEF-KAN-CHL-FLO | blaCTX-M-65, aac(3)-IV, aph(4)-Ia, aph(3')-Ia, tet(A), sul1, dfrA14, floR, cmlE | + |
| QT133 | Infantis | AMP-A/C-GEM-TET-CAZ-SF-SPE-DOX-NAL-SXT-CEF-KAN-CHL-FLO | blaCTX-M-65, aac(3)-IV, aph(4)-Ia, aph(3')-Ia, tet(A), sul1, dfrA14, qnrD1, floR, cmlE | + |
| QT134 | Infantis | AMP-A/C-TET-CL-SF-OFX-NAL-SXT | blaOXA-1, blaCTX-M-65, aac(6')-Ib-cr, tet(A), tet(C), tet(J), sul2, dfrA14, dfrA32, qnrD1, qnrD2, floR, catB, cmlE | + |
| QT143 | Infantis | AMP-TET-SF-SPE-DOX-NAL-SXT-CEF-KAN-CHL-FLO | blaCTX-M-65, aac(3)-IV, aph(4)-Ia, aph(3')-Ia, tet(A), sul1, dfrA14, floR, cmlE | + |
| JT81 | Indiana | AMP-A/C-TET-SF-NAL-SXT | blaTEM, blaNDM-5, blaCTX-M-14, aac(6')-Iy, aph(3'')-Ib, aph(6)-Id, aadA2, aph(3')-Ia, aac(6')-Il, aph(4)-Ia, tet(A), sul1, sul2, dfrA12, floR, cmlE | + |
| JT83 | Indiana | AMP-A/C-TET-CAZ-SF-ENR-OFX-DOX-NAL-SXT-CEF-CHL-CIP-FLO | blaOXA-1, blaCTX-M-55, aph(6)-Id, aph(3'')-Ib, aac(6')-Ib-cr, aph(4)-Ia, aadA5, tet(A), sul1, dfrA17, OqxA, OqxB, floR, catB, cmlE | + |
| JT84 | Indiana | AMP-A/C-GEM-TET-CAZ-CL-SF-ENR-OFX-DOX-NAL-SXT-CEF-CHL-CIP-FLO | blaOXA-1, blaCTX-M-55, aph(3'')-Ib, aph(6)-Id, aph(4)-Ia, aac(6')-Ib-cr6, aadA5, tet(A), sul1, dfrA18, OqxA, OqxB, floR, catB3, cmlE | + |
| JT85 | Indiana | AMP-A/C-GEM-TET-CAZ-CL-SF-ENR-OFX-AMK-DOX-NAL-SXT-CEF-KAN-CHL-CIP-FLO | blaOXA-1, blaCTX-M-55, aac(6')-Ib-cr6, aph(4)-Ia, aph(3'')-Ib, aph(3')-Ia, aph(6)-Id, aadA5, tet(A), sul1, dfrA17, mcr-1, OqxA, OqxB, floR, catB3, cmlE | + |
| JT86 | Indiana | AMP-A/C-GEM-TET-CAZ-CL-SF-ENR-OFX-DOX-NAL-SXT-CEF-KAN-CHL-CIP-FLO | blaOXA-1, blaCTX-M-55, aph(6)-Id, aph(3'')-Ib, aph(4)-Ia, aac(6')-Ib-cr6, aadA5, tet(A), sul1, dfrA17, OqxA, OqxB, floR, catB3, cmlE | + |
| LY93 | Indiana | AMP-A/C-TET-CAZ-SF-SPE-OFX-AMK-NAL-SXT-CEF-KAN-CIP-FLO | blaCTX-M-123, blaTEM , aph(4)-Ia, aadA16, rmtB, aph(3'')-Ib, aadA22, aph(6)-Id, sul1, sul2, dfrA27 | + |
| LY95 | Indiana | AMP-A/C-GEM-TET-CAZ-CL-SF-SPE-OFX-DOX-NAL-SXT-CEF-KAN-CHL-CIP-FLO | blaCTX-M-55, aac(3)-IV, aph(4)-Ia, aadA5, aph(3'')-Ib, aadA22, tet(A), sul1, sul2, dfrA17, mcr-1, floR, cmlE | + |
| LY94 | Kentucky | AMP-A/C-GEM-TET-CAZ-SF-SPE-OFX-DOX-NAL-SXT-CEF-KAN-CHL-CIP-FLO | blaTEM, aadA7, aph(4)-Ia, aac(3)-IV, tet(A), sul1, dfrA14, floR | + |
| QY104 | Kentucky | AMP-A/C-GEM-TET-CAZ-CL-SF-ENR-OFX-DOX-NAL-SXT-CEF-KAN-CHL-CIP-FLO | blaCTX-M-55, blaTEM, aadA7, aph(4)-Ia, aac(3)-IV, tet(A),sul1,qnrS9, qnrS1, floR | - |
| QY106 | Kentucky | TET-CL-SF-DOX-SXT-CEF-KAN-CHL-CIP-FLO | aac(6')-Ib-cr, tet(A), sul1, dfrA27, qnrB6, floR | + |
| QY110 | Kentucky | AMP-A/C-GEM-TET-CAZ-SF-ENR-OFX-AMK-DOX-NAL-SXT-CEF-KAN-CHL-CIP-FLO | blaCTX-M-55, blaTEM, aadA7, rmtB, tet(A), sul1, floR | - |
| QY112 | Kentucky | A/C-GEM-TET-SF-ENR-OFX-AMK-DOX-NAL-SXT-KAN-CHL-CIP-FLO | aph(4)-Ia, aac(3)-IV, aadA7, tet(A), sul1 , qnrS12, floR | - |
| QY113 | Kentucky | AMP-A/C-GEM-TET-CAZ-CL-SF-ENR-OFX-AMK-DOX-NAL-SXT-CEF-KAN-CHL-CIP-FLO | blaCTX-M-55, blaTEM, aac(6')-Iaa, aadA7, aac(3)-Id, aac(3)-IId, aac(3)-IV, aph(3')-Ia, rmtB, tet(A), sul1, floR | - |
| QY114 | Kentucky | AMP-A/C-GEM-TET-CL-SF-ENR-OFX-DOX-NAL-SXT-CEF-KAN-CHL-CIP-FLO | aadA7, rmtB, tet(A), sul1, qnrS12, floR | - |
| QY116 | Kentucky | AMP-A/C-GEM-TET-CAZ-SF-SPE-ENR-OFX-DOX-NAL-SXT-CEF-KAN-CHL-CIP-FLO | blaCTX-M-55, blaTEM, aadA7, aac(3)-IV, aph(4)-Ia, tet(A), sul1, floR | - |
| TY89 | Thompson | AMP-A/C-GEM-TET-SF-SPE-ENR-OFX-DOX-SXT-CEF-CHL-FLO | blaOXA-10, blaCTX-M-65, aac(3)-V, aph(4)-Ia, tet(A), sul3, dfrA14, qnrS1, floR, cmlE | + |
| QY103 | Thompson | AMP-A/C-GEM-TET-CAZ-SF-SPE-ENR-OFX-DOX-NAL-SXT-CEF-KAN-CHL-CIP-FLO | aph(6)-Id, aadA22, tet(A), sul3, dfrA14, floR, cmlA, cmlE | + |
| LY90 | Kedougou | AMP-A/C-GEM-TET-CAZ-SF-SPE-OFX-DOX-NAL-SXT-CEF-KAN-CHL-CIP-FLO | blaCTX-M-55, aph(6)-Id, aadA22, tet(A), sul3, dfrA14, qnrS1, floR, cmlA, cmlE | + |
| QY108 | Mbandaka | AMP-A/C-GEM-CAZ-CL-SF-ENR-O-FX-DOX-NAL-SXT-CEF-KAN-CHL-CIP-FLO | blaCTX-M-55, aac(3)-IV, aph(4)-Ia, sul1, qnrS9, qnrS12, floR, cmlE | - |
| QT129 | Brancaster | AMP-A/C-TET-CL-SF-OFX-DOX-SXT-CEF-KAN-CHL-FLO | blaTEM-176, tet(A), sul1, dfrA14, floR, cmlE | + |
| QT131 | Ohio | AMP-A/C-GEM-TET-CAZ-CL-SF-SPE-ENR-OFX-DOX-NAL-SXT-CEF-KAN-CHL-CIP-FLO | blaCTX-M-65, aac(3)-IV, aph(4)-Ia, aph(3')-Ia, tet(A), sul1, dfrA14, floR, cmlE | + |
| QT135 | Lexington | AMP-A/C-SF-SPE-SXT-CEF-KAN-CHL-FLO | - | - |

(-) = Not detected; (+) = Detected
